# The effect of practice and musical structure on pianists’ eye-hand span and visual monitoring

**DOI:** 10.16910/jemr.16.2.5

**Published:** 2023-05-29

**Authors:** Michel A. Cara

**Affiliations:** Pontificia Universidad Católica de Valparaíso

**Keywords:** eye-hand span, sight-reading, glances at the keyboard, working memory, musical expertise, eye-tracking, music reading, eye movement, individual differences

## Abstract

This study examines short-term improvement of music performances and oculomotor behaviour during four successive executions of a brief musical piece composed by Bartók, “Slovak Boys’ Dance”. Pianists (n=22) were allowed to practice for two minutes between each trial. Eye-tracking data were collected as well as MIDI information from pianists’ performances. Cognitive skills were assessed by a spatial memory test and a reading span test. Principal component analysis (PCA) enabled us to distinguish two axes, one associated with anticipation and the other with dependence/independence on written code. The effect of musical structure, determined by the emergence of different sections in the score, was observed in all the dependent variables selected from the PCA; we also observed the effect of practice on the number of fixations, the number of glances at the keyboard (GAK) and the awareness span. Pianist expertise was associated with fewer fixations and GAK, better anticipation capacities and more effective strategies for visual monitoring of motor movements. The significant correlations observed between the reading span test and GAK duration highlight the challenge of working memory involvement during music reading.

## Introduction

Music reading is a fundamental competence for most western musicians, in which the role of knowledge-based expectations is decisive (60). Music reading is considered a complex, multitask activity that requires the musician to handle different kinds of information efficiently, decode the written text, and simultaneously transform information into motor movements (4). Music reading without involving motor outputs is defined as silent reading. In the present research, music reading was studied mainly in relation to specific motor outputs.

Different cognitive skills are involved in music reading, such as mental speed (47) and working memory (11; 58; 63). The working memory model (8) consists of a multi-component system that temporarily stores and processes speech and sound (phonological loop) and visual information (visuospatial sketchpad) in complex cognitive activities. A central executive system with limited capacity controls both subsystems, while an episodic multidimensional buffer permits the different subsystems to interact with one another and with the long-term memory (7). Ockelford (63) proposes the existence of a domain-specific executive module with a variety of functions such as “track and direct the musical narrative simultaneously at different architectonic and hierarchical levels to ensure longer-term coherence” (p. 29).

Sight-reading, i.e. performing music from a score at first sight and without preparation, is a crucial competence for musicians. The tonal and textural organization of the music influences sight-reading by engaging bottom-up and top-down processes concurrently (54). It has been stated that visuospatial cognition in musicians should be enhanced by practicing sight-reading (77) or by long-term musical practice (10; 38; 46). More recently, Aiba and Sakaguchi (4) pointed out the link between music reading and the capacity of processing geometrical features in skilled pianists. In fact, fluency in music reading depends on effective anticipation of motion (36) or the notes to be played during reading (32; 76; 79; 89) and these anticipation capacities seem to be related to selective mobilization of cognitive resources.

### Eye-Hand Span

The study of eye movements with eye-tracking devices has improved our understanding of the anticipation process during music reading by measuring the eye-hand span (EHS), defined as the distance between the performed note and the actual fixation point in the score. During this time, information is stored and processed in the working memory and motor acts are programmed. Early works showed that musicians can anticipate up to 7 notes and that EHS is an elastic measurement that varies in relation to phrase boundaries (75, 76). Different musical textures can affect measurement of the EHS (85), as can melodic structures (66; 89). The EHS has been measured in terms of time (30; 89), number of notes ( 71; 76; 89) and beats ( 55; 66). The EHS in terms of time or “time index” appears to be about 1 s (89) and can be affected by tempo (72).

A short period of practice does not appear to increase the EHS (13; 72). The complexity of the music can reduce the size of the EHS (32; 55; 72; 81; 89). Previous studies have also reported that expert musicians present longer EHS (30; 32; 81). Lim et al. (55) claimed that the EHS, more than a sight-reading predictor, is an indicator of the strategies adopted by musicians to cope with musical complexity. For further, recent references on EHS see Perra et al. (67).

### Expertise and Eye Movements

As mentioned previously, expert music readers have better expertise-associated capacities. These may be linked to many factors, such as more efficient mobilization of visuospatial capacities (see above), the capacity to elaborate cross-modal representations (24), their “chunking abilities” (45; 69; 84; 88) or even their ability to imagine sounds during reading (51).

Expertise has also been associated with shorter duration and smaller numbers of fixations (22; 84) which are related to more efficient decoding and retrieval of information capacities, enabling experts to reduce the time spent on the acquisition of visual information. Thus, from the perspective of Long-Term Working Memory (LTWM) theory (27), expert musicians show more independence from the written code (the score) as their music reading competence relies on the capacity to elaborate representations where the encoding process is not modality-dependant (amodal hypothesis), based on their previous knowledge (23). Moreover, the mechanisms underlying the number:duration ratio of eye fixations seem to depend mostly on the structure of the music (37). Although the model proposed by Ericsson and Kintsch (27) should account for skill acquisition, retrieval structures seem to be insufficient to explain memory improvement indirectly associated with achieving expertise (35).

In piano music reading, an essential skill concerns the efficiency of the visual monitoring of motor movement (in this study, glances at the keyboard, GAK). A GAK is a quick movement of the eyes, sometimes accompanied by a head movement, necessary to obtain feedback from the keyboard or hands while reading music or playing by ear. In fact, GAK are necessary when specific requirements – such as two-hand coordination, displacements, tessitura changes or hand-crossings – are frequent. GAK can also constitute a handicap if the musician is unable to coordinate the manoeuvre in real time. Thus GAK could, in some cases, disrupt the musical flow or the encoding process (52). Land and Furneaux (48) suggested that GAK can be considered a multitask activity, where motor skill learning must be accompanied by particular eye movement instructions. Previous research has found that the frequency of GAK is lower among more experienced pianists (32; 49; 52), and that more skilled pianists are able to look at the keyboard more quickly than less skilled pianists (13).

### Music Structure and Stylistic Features

There is evidence that music encoding depends on music structure – the form of a musical sequence implicitly determines its limits in each individual, and the length must be suited to working memory capacities (21; 50). Moreover, it has been proposed that the extraction of structural regularities is important in processing and enjoying music (28; 62). There is also evidence that music structure is processed in language areas of the brain (53), and that structural processing overlaps between music and language (28). Moreover, structural cues in music are critical for organizing practice and as a basis for recalling musical information in the exploration of a musical piece, in both the instrumental (14; 16; 39; 86) and vocal domains (33; 34).

Concerning music reading, so far as we know little attention has been devoted to analysing eye movements and the cognitive skills involved when reading contemporary music (56; see also 70). Works in this field have mostly been oriented towards manipulating stylistic features (complexity) and studying their effect on eye movements – for example, with Beethoven piano pieces (74) – or towards comparing anticipation behaviour when reading contrasting violin pieces of Telemann and Corelli (89); or even studying the impact of complexity when it is manipulated in brief pieces specially composed for an experiment (55).

### Rationale for the Study

Although, as mentioned above, there are various eye-tracking studies in the context of sight-reading, few works have systematically studied the early stages of the learning process immediately after the musician's first contact with the score. In our view, this initial process is decisive for the success of the musical interpretation of a given piece of music. Thus, the first focus of analysis in the present study is on short-term improvement in reading and performing a new piece of music.

Much of the knowledge about anticipation in music reading has been built on non-ecological stimuli, very often based on a given tempo (see 68). Ecological material generally presents thematic development, making it possible to explore the learning process by focusing on musical structure. For this reason, the second focus of our analysis is on the effects of musical structure on music performance and anticipation. We believe that if no determined tempo is imposed, we can study the learning process in a more naturalistic situation.

The above factors are addressed considering performance, eye-movements, and anticipation measures; special attention is paid to study of anticipation mechanisms and visual monitoring of motor movements. In fact, there is currently very little research into visual monitoring of motor movements, even though the technology to study this area exists. Another important aspect of the present work, therefore, is related to the study of GAK, as well as anticipation features related with visual monitoring of motor movements, based on Baddeley’s model (5, 6; 31). The literature on the development of these concepts is still incipient.

Finally, it is important to note that understanding of expertise-related variables is crucial to draw conclusions regarding both skill acquisition and the involvement of cognitive skills in learning. Thus, the third focus of this work is on studying the association between expertise and cognitive skills through the different variables studied. We fully adhere to the Engeström expansive learning approach—in which learning and skill acquisition form a continuous process where boundaries are reconstructed permanently in the activity (25; 26). Our approach is to understand how musicians advance through these limits. To address this issue, we will evaluate different expertise approaches: one focusing on academic achievement (comparing undergraduate vs professional musicians) and a task-related approach where expertise groups are created based on performance results.

### Hypotheses

Structural cues in music are critical for organizing practice and for achieving the ongoing encoding processes. Music reading is a complex, multitask activity and visual features affect eye-movement parameters. From this perspective, thematic development should then increase the cognitive load, affecting working memory capacity for the momentary storing and processing of musical information.

Thus, in response to the first and second focus of the experiment we expected:

(1) From a multivariate approach, anticipation variables sufficiently represented and associated with expertise.
(2) An effect of practice on the variables studied (eye-movement, and anticipation measures). We expect to find a decrease in the number and duration of fixations and the number and duration of GAK. In accordance with previous studies (see Introduction), no effect of practice on anticipation measures (EHS and awareness span) is expected.
(3) An effect of music structure on eye-movement, and anticipation measures. In those sections presenting thematic development, we should observe an increase in the number and duration of eye fixations, a decrease in the anticipation measures and an increase in the number of GAK.

Turning to the third focus of the study, we expected that pianist expertise would be associated with fewer fixations and GAK (less dependence on visual feedback); longer EHS and awareness span; and shorter GAK. We expected that the two expertise approaches addressed in this study should complement one another when pianists’ learning strategies were analysed. In the area of cognitive skills, addressed by Baddeley’s model, we expected to find a strong association between short-term spatial memory and EHS, implying reliance on the visuospatial sketchpad for music reading.

## Method

### Participants

Twenty-two pianists participated in the experiment; their average age was 30 years (*SD* = 14.19) and their piano experience ranged from 9 to 54 years. The panel consisted of 10 professional and 12 undergraduate pianists (in the last three years of conservatory). Pianists were active musicians pursuing their careers, mainly in Paris and Dijon, and they received payment for their participation in the study. All participants signed an informed consent before the experiment.

### Apparatus

A Tobii T120 eye-tracker with a sampling frequency of 60 Hz was used to monitor eye movements. The pianists played a MIDI piano (Yamaha SX 100 HQ) connected to a Digidesign M-Box 2 interface. The Tobii T120 screen was recorded with a video camera. A video editing program (Adobe Premier Pro CS5) enabled the MIDI and eye-tracker signals to be synchronized. The distance between the screen and the pianist’s head was approximately 600 mm. No chin rest was used because the pianist needs to perform natural head movements for GAK. The algorithm used for the raw data analysis was provided by the Tobii Studio software. The definition used for a fixation was a gaze maintained within a 50-pixel radius for at least 100 msec. Analysis was based on the ‘‘normal’’ ClearView validity filter averaged across both eyes. The fixation index was given by the software; fixations that exceeded 2000 msec were excluded from the data, as recommended by Holmqvist et al. (40).

### Stimulus

**Figure 1.**
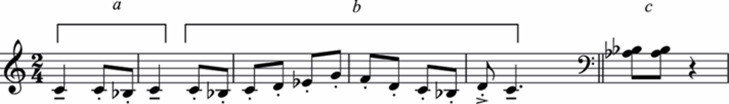
The main motifs that characterize this piece by Bartók, from Ten Easy Pieces, Sz 39 (1908).

The musical stimulus presented to participants was Bartók’s "Slovak Boys’ Dance”, a bimodal piece (Dorian and Aeolian modes), whose main theme (five bars) is divided into two main motifs: "a" and "b". An additional motif "c" plays an important structural role and accompanies the theme (see Figure 1). This structural role is essential for thematic development, since it leads to the reformulation of a main theme that is contrasted using different constructive procedures.

The music was scanned and converted to JPEG format. The variations of the two motifs serve as a base for thematic development. The image dimensions were 599 × 841 px with 72 px/inch resolution. On average the note head size was 0.25 cm, the bar width was 3.49 cm, the bar height 2.72 cm, and the distance between notes (quavers) 0.86 cm.

 In the musical piece the difficulty increases principally in the central sections (i.e. 2 and 4) linked with thematic development. The first section (measures 1-10) is characterized by the introduction of the theme in the right hand and then in both hands. In the second section (measures 11-22), motif “c” appears, giving rise to the first thematic development by alternation with appearances of the main motifs. The same happens in Section 4 (measures 30-45). The last section (measures 45-54) is characterized by an ostinato in the left hand accompanying the main theme. All the participants declared that they were unfamiliar with the piece.

Visual features of the musical material are closely related with structural features. We can in fact distinguish some of the main elements: gradual melody (main theme in section 1); gradual melody interrupted with jumps (variations of the main theme in section 2 and 4, accompanied by motif “c”); repetition of motif “a” accompanied by motif c (section 3) and with an ostinato (section 5). (For the full score, see Appendix.)

Those sections where the musical material is developed were considered more demanding for the participants. These assumptions were confirmed by external evaluation of four experienced pianists (one piano teacher from the Universidad de Chile, one from the Pontificia Universidad Católica de Valparaíso and two teachers from the Instituto Superior del Profesorado de Música Lilia Yolanda Perenno Elizondo of Argentina) who were asked to evaluate the difficulty of each section from 1 to 10. Results were as follows: Section 1 (2.75); Section 2 (5.25); Section 3 (4.5); Section 5 (4.25). The teachers were asked to evaluate the difficulty of learning the piece in a short session or by sight-reading. They were also asked to consider the difficulty that an experienced pianist might experience.

### Corsi Block-Tapping (CBT) Test

The CBT (17; 59) was applied to measure short-term memory using recall of sequential locations. The instructions and materials were displayed on the computer screen.  Each trial began with a 5-second countdown. A total of 10 figures, 20 × 15 cm each, were presented on the screen.  In each sequence, blocks were highlighted in random order. Upon the completion of a sequence, the participants reproduced the order they recalled by clicking on the “blocks” with the mouse. Initially, the sequences contained only two items to recall (training). To advance to the next level, at least one of the total of three presentations had to be successfully reproduced. After three sequences of the same length, the number of items to recall was increased by one until the limit of the participant’s capacity was reached. The dependent variables taken into account were the total number of correctly reproduced items in the correct serial position. Points were awarded for each correct item (order and location) regardless of whether the sequence had been reproduced in its entirety.

### Reading Span

The working memory assessment included both the storage and processing capacities of the participants. The French version (20; 61) of the traditional Daneman and Carpenter (19) Reading Span Task (RST) was applied. It included true/false statements (second version). The participants were required to read sentences in French, and at the same time they had to remember a number (storage component).  The participants had to complete a training block (three sequences with two extracts each) followed by 30 test phrases. These test phrases consisted of a total of 12 sequences which were divided into blocks of 9, 12 and 20 phrases. The participants had to press "o" on the keyboard for "yes" to indicate that the sentence was consistent and "n" if not (processing component). Pressing “o” or “n” would allow the participant to view the next sentence. To adopt a continuous measure, the total number of digits recalled and the total number of correctly reported true/false statements were taken into account.

### Design and Procedure

CBT was applied immediately before the sight-reading session. A calibration phase with the eye-tracker device preceded each trial. After this calibration, pianists were instructed to start playing while the score was shown on the screen; they were required to play almost immediately, as previous study of the piece before the first trial was not allowed. The participants chose their own tempo based on their own sight-reading skills. The score was composed to be displayed on two pages; however, for the purpose of this study, one page was visible at a time. At the same time, the researcher controlled the display of the pages using a second monitor (Live Viewer in Tobii studio). When musicians reached the end of the score, the researcher changed the page. The participants performed the musical piece four times, with two minutes of practice between trials, and had to read both hands. The RST was administered on completion of the sight-reading session.

### Data Analysis

Matlab programming, developed by the researcher, was used for data analysis. After the temporal and/or spatial distance between the fixation and the performance points had been determined, the EHS was calculated for each note (n = 307) and each fixation. The nearest point (fixation) to a given note in the score was taken into consideration for the same timestamp of both MIDI event and eye-tracker information. 

Notes belonging to the same chord were considered to belong to the same unit. Particularly the piano (or other polyphonic instruments), require different measures of the EHS due to their harmonic and polyphonic textural possibilities. In fact, the method used to measure the EHS should be linked with the musical material (see 68). Due to the differences between musical organization and texture, the following EHS measures were used: (1) eye-hand span in quavers (EHSQ), depicting beat subdivisions (four quavers per bar), 213 data points; (2) eye-hand span in beats (EHSB), 108 data points; and (3) eye-hand span in notes (EHSN), totalling the number of notes from the beginning of the performance until the fixation point, 307 data points. Considering that the most frequent rhythm in the score is the quaver, the EHSQ represents the main unit of reference. The EHSB is closely related with harmonic fluctuations or vertical units (chords).

To calculate the variability and establish a minimum and maximum threshold for data analysis of GAK we analyzed the raw data from Tobii studio in one hundred simulations. The software sends specific validity codes when the infrared sensors fail to reflect the cornea. The average values of the simulations ranged between 360 ms and 980 ms. Gilman and Underwood (32) deemed about 750 ms as the threshold for GAK.

Blinks were not systematically excluded from the data, given that Tobii Studio validity codes might underestimate blink duration (41). In the results, 15% of the data were between 100 ms and 300 ms, which implies that some measures might be inflated by the occurrence of blinks.

 The available data points for each participant depended on the number of fixations matched with a performance note. The average GAK and EHS values per section were calculated in order to perform the statistical analysis (ANOVA). The Matlab software algorithm was used for comparison of performance data between the pianists.

Several matrices were created for each performance. Some notes were removed, and others were added, using the error detection information provided by dynamicMatch MIDI Toolbox. However, MIDI parameters characteristic of each performance were preserved (i.e. onset and durations of the notes, MIDI pitch and keypress velocity). Three types of errors were considered to obtain the score (additions, substitutions, and deletions). In the case of the notes that appeared in the score and were not played (i.e. deletions), both the average and the onset times were calculated, retaining the proportion of the referential MIDI information. The dimensions of the corrected matrices were the same for each performance; thus, each matrix contained the same number of notes, which allowed comparisons between performances within sections of each musical score. Finally, each note of the corrected matrix was assigned the following: (1) a measure of the EHS (which is a non-continuous event as it requires both a note and a fixation point simultaneously); (2) a GAK measure (another non-continuous data type); (3) errors; and (4) tempo. Timing was calculated as the difference between the tempo measurement assigned for each beat.

**Figure 2.**
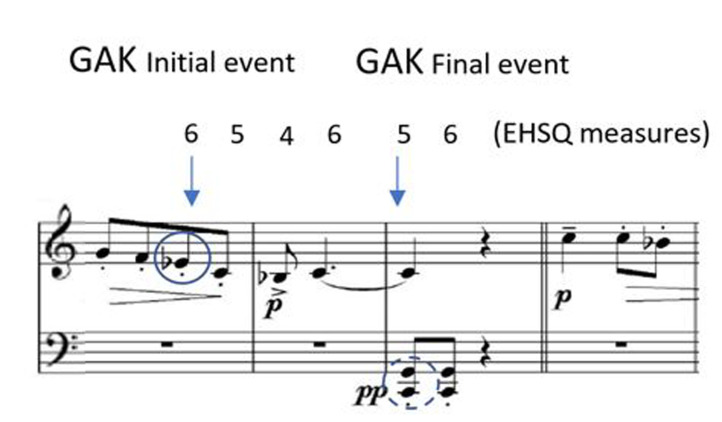
The awareness span calculation includes an initial event, or the distance between the performed note (Eb – in circle) and the first time that the pianist looked at a point in the score where subsequently he/she would need to look at the keyboard to perform it (C and G in the left hand – dotted circle), named the final event. All the measurements of the EHSQ were considered between these points (i.e. 5,4,6,5). For example, when the pianist is playing Eb (in circle), he/she is looking six quavers ahead.

The awareness span is conceived as an anticipation measurement (e.g. preparation for a difficult pianistic event); the starting point is the moment when the pianist looks at a certain note in the score that will subsequently need a GAK before it can be performed (for example, if it is far removed from the preceding notes). In other words, the awareness span provides an answer to the following question: Does the pianist increase anticipation before a GAK to prepare the necessary end-goal sequence of movements? To calculate the awareness span – understood as the interval (in quavers) between the participant’s fixation on a sensitive point in the score (the initial event of the measurement) and the glance at the keyboard a few milliseconds later corresponding to the same sensitive point (the final event of the measurement) – the average of all the available EHSQ measures in these specific intervals was used (see Figure 2).

Both GAK and EHSQ measurements (before each GAK) can have an impact on the awareness span estimation. However, not all pianists presented this dependence, and some exceptions were noted (2.95%). To deal with these exceptions, we grouped Section 2 with Section 4 and Section 3 with Section 5, as each pair presents similar musical material. We were then able to perform an ANOVA to evaluate the effect of music structure on the awareness span.

### Statistical Analysis

In the first place, to test the first hypothesis and to define the relationship between the variables studied, Principal Component Analysis (PCA) was carried out. PCA is an exploratory tool for data analysis that allows a reduction in dimensionality, through new variables or principal components, while preserving as much variability as possible (44). Secondly, to test our second and third hypotheses, a repeated measures analysis of variance (ANOVA) was performed on the variables selected from the PCA, with two within-subject factors (section and trial) and one between-subject factor (expertise).

For each participant, a mean value per section was calculated with the total available data points; for example, for fixation number, the total number of fixation points effected within each section. Thus, a total of 20 observations (5 sections x 4 trials) was necessary for each participant.

Concerning pianist expertise, for the first task-related approach, a score was calculated considering performance speed and accuracy, by subtracting the overall number of errors from the overall tempo. This score was included in the PCA as a continuous variable. For the second perspective (undergraduate/professional pianists) a categorical factor was included in each ANOVA.

## Results

When the data presented outliers exceeding two standard deviations from the mean in one or more sections, pianists were excluded from the analysis. This criterion was based on recommendations for single construct techniques (+- 2.5 *SD* from the mean) (2; 57) applicable in cases where there are viable arguments to consider those extreme values as invalid (see also 64 survey). One pianist had to be removed from the fixation duration (*M* = 622.68, *SD* = 199.24) analysis. Since there are no data for us to assess when the pianist does not glance at the keyboard (in one or more sections), four pianists were removed from the GAK duration analysis and one pianist was removed from the awareness span analysis. It is worth mentioning that in these cases the pianists always looked at the score and not at the keyboard, at least in one of the sections. Descriptive statistics for performance, eye-movements and anticipation measures are presented in Table 1.

**Table 1.** Performance measures, eye movement measures and anticipation measures – means, standard deviations and confidence intervals.

Measures	*n*	*M*	*SD*	95% CI	
				*LL*	*UL*
Fix N	22	14.6	6.35	14	15.19
Fix D	21	464.04	166.35	448.09	479
GAK N	22	4.64	3.03	4.35	4.92
GAK D	18	492.96	190.29	473.24	512.69
EHSQ	22	2.71	0.7	2.65	2.78
EHSB	22	2.47	0.66	2.4	2.53
EHSN	22	4.13	1.44	3.99	4.26
Aw.span	21	3.19	0.52	3.08	3.3
Tempo	22	131.04	22.88	120.89	141.19
Errors	22	20.28	12.38	14.78	25.76
Timing	22	16.91	6.05	14.23	19.59

Note. CI = confidence interval; Fix N = number of fixations; Fix D = fixation duration; GAK N = number of glances at the keyboard; GAK D = duration of glances at the keyboard; EHSQ = eye-hand span in quavers; EHSB = eye-hand span in beats; EHSN = eye-hand span in notes; Aw.span = awareness span

### Principal Component Analysis

 The mean score for all four trials was calculated for the following measures: (1) performance measures – tempo, timing and errors (full score considering additions, substitutions and deletions); (2) eye-movement measures – number and duration of fixations, number and duration of GAK; (3) anticipation measures – EHSQ, EHSB, EHS and (4) cognitive skills – CBT and RST. The PCA was based on a correlation matrix and the data were included without any culling or transformation (see Table 2).

Furthermore, in accordance with their representation on the factorial plane, the following variables were selected: number of fixations (Fix N); number of GAK (GAK N) duration of GAK (GAK D); EHS in quavers (EHSQ); and Awareness span (Aw.span). Tempo and cognitive skills (Corsi and RST) were included as supplementary variables, because of their weak representation in the factorial plane. Proficiency was also included as a supplementary variable, firstly because it is a composite variable and secondly because it was significantly correlated with tempo (*r* = .85, *p* = .001).

**Figure 3.**
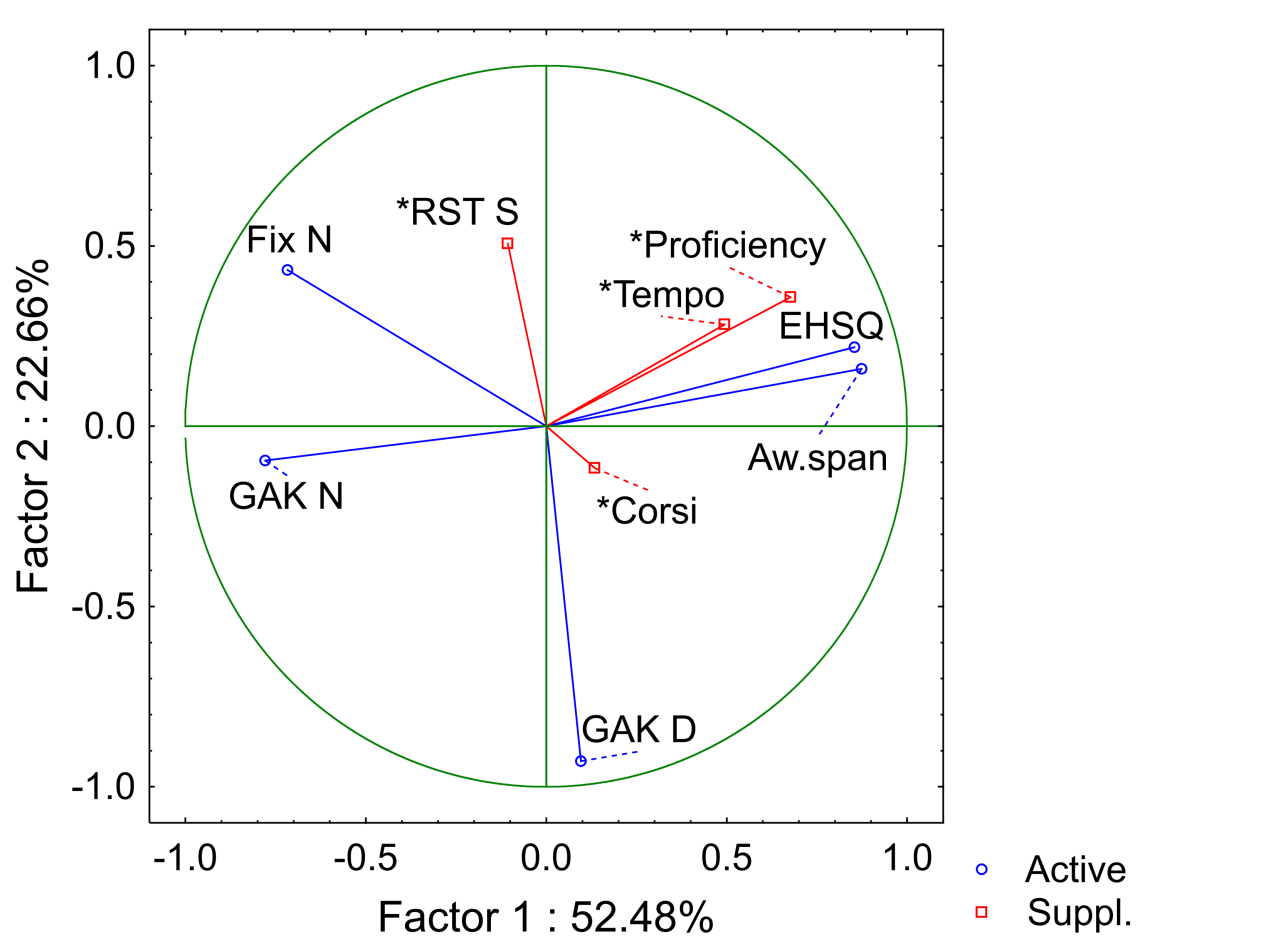
Principal component analysis. Active variables are used to compute the PCA. Supplementary variables are useful to observe the relationship among the complete set of variables, enriching the interpretation without influencing the barycentre. They are included after the analysis is performed (1). Vector length represents the contribution of each variable in establishing the Principal Components (the longer the vector, the more important the contribution).

Two principal axes could be distinguished from the PCA, opposing anticipation variables (EHSQ, Aw.span) with variables accounting for dependence on the written code, and the visual monitoring of motor movements (Fix N and GAK N). Indeed, these axes explain 75% of the variance (see Figure 3).

The PCA results suggest that anticipation should be fostered when strategies for information intake are more efficient; this should enable tempo increase in return. In fact, results seem to indicate that skilled pianists are able to reduce the acquisition of visual information as a part of the learning process through enhanced visual-kinaesthetic representations, particularly the number of fixations and GAK, in comparison with less skilled pianists. The association between proficiency and EHSQ or Awareness span might suggest more effective control of planning strategies in faster and more accurate pianists.

Moreover, the duration of GAK seems to be a compromise between the two poles, suggesting an independent implication of verbal working memory capacities in the process of learning a new given musical piece.

**Table 2.** Correlation matrix. Supplementary variables in italics.

	1	2	3	4	5	6	7	8	9
1. Fix N	1	.64***	-.28	-.37	-.37	-.13	.30	-.45*	-.30
2. GAK N	.64***	1	-.12	-.45*	-.50*	-.02	.11	-.77***	-.58*
3. GAK D	-.28	.12	1	-.01	.07	.14	-.48*	-.36	-.27
4. EHSQ	-.37	-.45*	-.01	1	.89***	.21	.01	.55**	.42
5. Aw.span	-.37	-.50*	.07	.89***	1	.06	.07	.47*	.34
*6. Corsi*	-.13	-.02	.14	.21	.06	1	-.39	.07	-.11
*7. RST S*	.30	.11	-.48*	.01	.07	-.39	1	-.09	.02
*8. Proficiency*	-.45*	-.77***	-.36	.55**	.47*	.07	-.09	1	85***
*9. Tempo*	-.30	-.58*	-.27	.42	.34	-.11	.02	.85***	1

Note. Fix N = fixation number; GAK N = number of glances at the keyboard; GAK D = duration of glances at the keyboard; EHSQ = eye-hand span in quavers; Aw.span = awareness span; Corsi = Corsi block-tapping test; RST S = reading span storage. *p < .05; **p < .01; ***p < .001.

In brief, PCA analysis led us to select some crucial variables explaining 75% of the variance. In the next section, the effect on these variables of the structure of the music and the practice will be addressed.

### Selected Variables Analysis

To assess the effect of musical structure and practice on the selected dependent variables determined by the previous PCA analysis, a repeated measures ANOVA was performed for each variable. As we intended to test predefined hypotheses (confirmatory purposes), it was unnecessary to adjust the level of α (18); similarly, if conjunction status is granted in Hypothesis 2 (see 73). However, bearing in mind that different tests were considered in the same experiment, we applied an adjustment to the overall Type I error probability (Bonferroni α adjustment of 0.05/5), as suggested by Huberty and Morris (42). Despite the evidence of the effect of tempo on eye movement measures (67; 72), this variable is not included as a covariable given its weak representation in the factorial plane.

#### Musical structure

**Figure 4.**
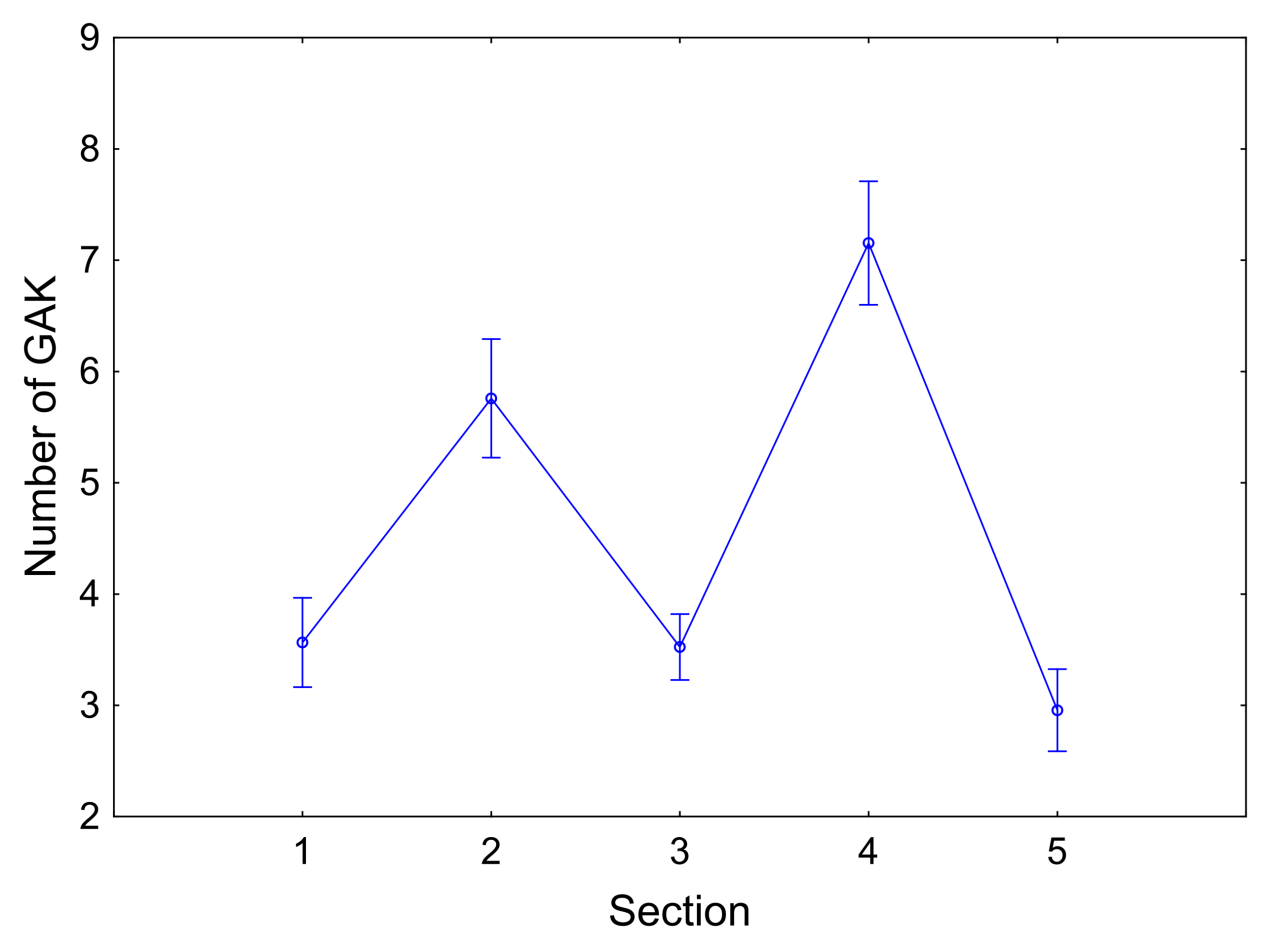
Differences between sections relative to the number of GAK. Sections 2 and 4 present thematic development. Error bars show the standard error of the mean.

Musical structure affected all the selected variables (see Table 3). As expected, the structure of the music had an effect on eye fixations, as the number of fixations increased with thematic development. In the last section in particular, as the passage was repetitive, the number of fixations decreased. During the thematic development (Sections 2 and 4), the number of fixations did indeed increase and GAK duration tended to decrease, while the number of GAK increased, particularly in Section 4 (see Figure 4). However, in the last section (ostinato), both the duration and number of GAK decreased. Anticipation increased with thematic development, which included the EHSQ (see Table 4).

**Table 3.** Repeated Measures ANOVA summary for eye movement measures and anticipation measures.

	Musical structure (section)			Practice effect (trial)			Expertise		
Measures	*df*	*F-*value	η_p_^2^	*df*	*F*-value	η_p_^2^	*df*	*F*-value	η_p_^2^
Fix N	4, 80	101.66***	.835	3, 60	9.02***	.310	1, 20	0.93	.044
GAK N	4, 80	43.34***	.684	3, 60	4.42 **	.181	1, 20	1.95	.089
GAK D	4, 64	4.96**	.236	3, 48	1.24	.066	1, 16	3.62	.018
EHSQ	4, 80	5.48***	.215	3, 60	3.39 (*)	.145	1, 20	2.02	.092
Aw.span	2, 38	4.31 (*)	.185	3, 57	3.95**	.172	1, 19	1.19	.059

Note. d.f. = degrees of freedom; Fix N = number of fixations; GAK N = number of glances at the keyboard; GAK D = duration of glances at the keyboard; EHSQ = eye-hand span in quavers; Aw.span = awareness span. Significance indication in parentheses means that significance was not reached after Bonferroni correction. *p < .05; **p < .01; ***p < .001.

#### Practice effect

The effect of practice was observed in three of the selected variables (see Table 3). Pianists decreased the number of fixations and the number of GAK throughout the trials, with a non-significant increment in the last trial. They were thus able to reduce the acquisition of visual information as a part of the learning process through enhanced visual-kinaesthetic representations (see Table 4).

**Figure 5.**
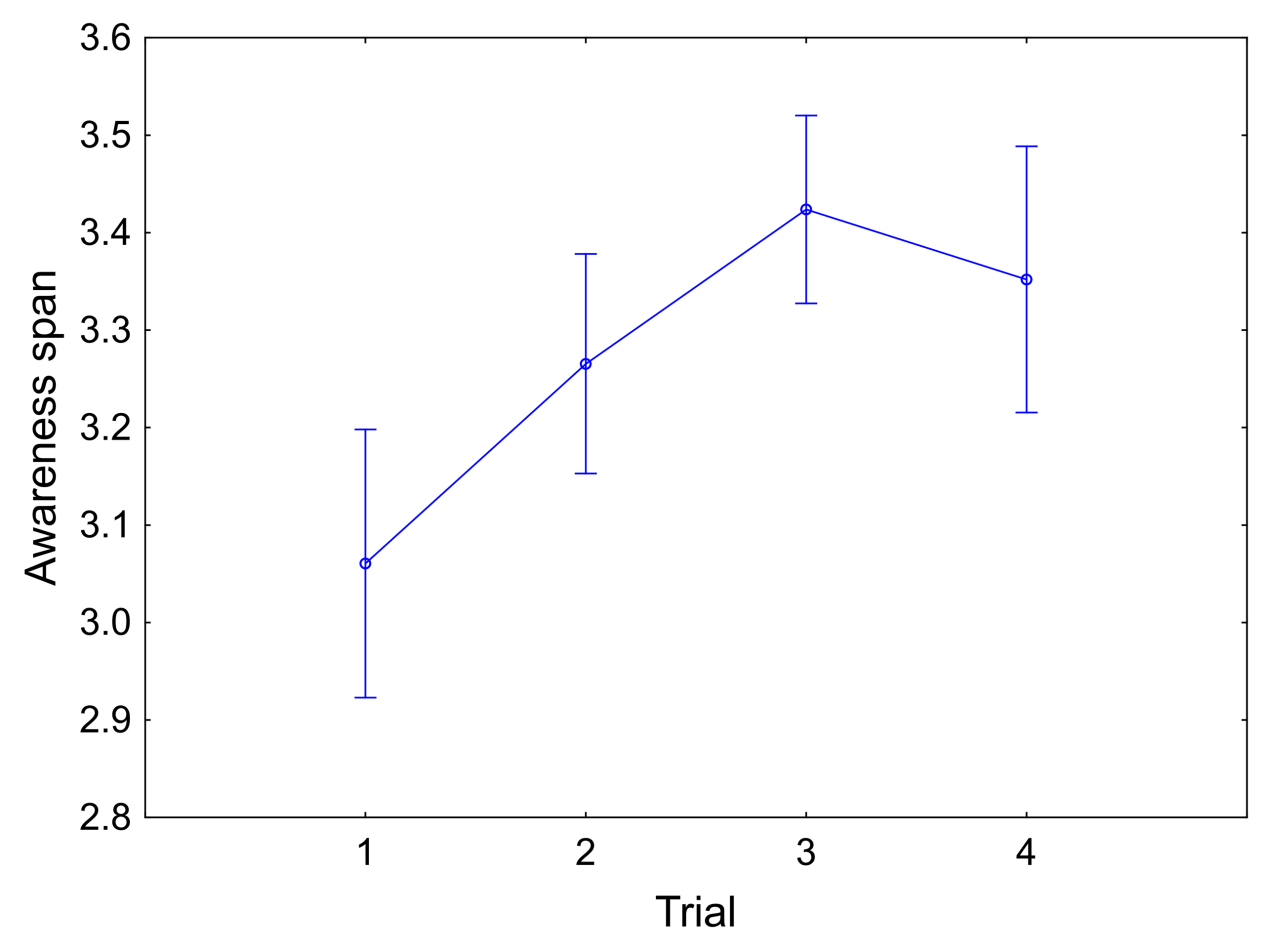
Progress of awareness span across the trials. Error bars show the standard error of the mean.

A significant improvement in the awareness span across the trials was found. In the third trial, this span increased at higher values while the number of GAK became constant (see Figure 5).

#### Expertise and cognitive skills

As stated previously, a score to measure task-related expertise (proficiency) was calculated by subtracting the overall number of errors from the overall tempo. The second approach separated the two groups: professionals and undergraduate pianists. The implications of the first score were presented in the PCA analysis. The second approach was addressed in the subsequent ANOVAs, where we found no effect of expertise on the selected variables, suggesting that the two approaches are complementary and non-exclusive (see Table 3).

In cognitive skills, negative correlations were found between RST (storage) and GAK duration. In turn, shortterm spatial memory was not correlated with the EHSQ or other variables (see Table 2). RST processing was not correlated with any of the variables studied.

**Table 4.** Post-hoc analysis (Tukey HSD), showing mean values and probabilities of section and trial factors for each measure.

Measures	Section 1	Section 2	Section 3	Section 4	Section 5	Trial 1	Trial 2	Trial 3	Trial 4
Fix N	12.45 b (4.5)	15.45 c (4.92)	12.42 b (4.08)	22.4 d (5.7)	10.27 a*** (4.36)	16.41 b (6.29)	14.1 a (6.1)	13.89 a (6.19)	14 a*** (6.54)
GAK N GAK D	3.59 a (2.28) 541.05 b (212.86)	5.85 b (3.03) 482.3 ab (160.62)	3.53 a (1.92) 528.45 b (186.05)	7.24 c (3.18) 489.41ab (127.35)	2.98 a*** (2.18) 423.6 a** (229.17)	5.27 b (3.44) 507.24 (160.58)	4.62 ab (2.91) 517.30 (224.67)	4.27 a (2.84) 464.15 (165.78)	4.39 a** (2.84) 483.16 *ns* (201.44)
EHSQ	2.63 a (0.64)	2.69 ab (0.54)	2.5 a (0.67)	2.97 b (0.69)	2.78 ab*** (0.84)	2.56 (0.69)	2.7 (0.68)	2.85 (0.71)	2.74 ab(*) (0.68)
Aw.span	3.46 a (0.89)	3.21 ab (0.60)		3.11 c(*) (0.74)		3.01 a (0.55)	3.12 ab (0.52)	3.39 c (0.46)	3.26 bc** (0.49)

Note. Letters (a, b, c or ab) were assigned to distinguish the groups (ANOVA Tukey groups). Where there was no significant difference between groups (in sections or trials), no grouping letter was added. *SD*s appear in parentheses. Fix N = fixation count; GAK N = number of glance at the keyboard; GAK D = duration of glances at the keyboard; EHSQ = eye-hand span in quavers; Aw.span = awareness span. ns = not significant. *p < .05; **p < .01; ***p < .001.

## Discussion

Improvement in performance of a new piece for piano in a short session was examined by considering performance, eye movements and anticipation measures (EHS and awareness span). It was observed overall that these variables vary according to factors related to the structure of the music (sections), which confirms our second hypothesis, and with practice (trials), which partially confirms our third hypothesis as the effect was observed in 60% of the variables. No significant differences were found between professionals and undergraduate pianists; however, task-related expertise (proficiency) was particularly associated with fewer GAK and fixations, as well as larger EHSQ and awareness span, which partially confirms our third hypothesis. On the other hand, the representation of the EHS in the factorial plane and its association with expertise-related variables confirms our first hypothesis.

Contrary to our hypothesis related to cognitive skills, no association between short-term spatial memory and anticipation capacities was observed, opening a debate in relation to the mobilization of these capacities according to the type of music stimulus.

### Cognitive Resources

Slovak Boys’ Dance shows the influence of folk music in certain of Bartók’s pieces. The closeness to the vocal dimension as well as the musical form and structure involve the implementation of particular learning strategies. In fact, in contrast to our previous findings, we did not observe visuospatial reliance, thus our related hypothesis was not confirmed. Selective reliance on visuospatial capacities has been reported in music reading (4; 13) and in other domains linked with physiological arousal or cognitive effort (78). Moreover, Williamson et al. (87) argue that musicians would have the ability to create and maintain music by using a variety of codes, e.g. visual, auditory and tactile. From this perspective, and considering the technical requirements and the stylistic features of the musical stimulus, different reading strategies were required, implying the mobilization of different cognitive resources.

Related to this issue, another important finding in our present report concerns the implications of the oculomotor system for multitasking activities, highlighted by correlations between the RST and the duration of GAK. This suggests the mobilization of common processes (19; 82), and highlights the role of working memory capacities in the efficiency of multitask activities. For visual feedback, pianists must be able to share attentional resources between two tasks (decoding the score and searching for information on the keyboard). This multitasking activity is comparable to conducting music, driving or typewriting. Perhaps the most notable finding are the significant correlations between the RST and GAK duration. This suggests, from the point of view of the time-based resource-sharing (TBRS) model of working memory (9), that in music reading the ability to change between tasks, or to switch between storage and processing activities, seems to depend on expertise. Moreover, the difficulty of the task is then reflected in the cognitive load as an increase in the resources used, resulting in a decrease in resources dedicated to maintenance activities (83); this is consistent with the assumption that different maintenance mechanisms (other than sub-vocal rehearsal) must be involved in working memory maintenance (see 12). Our results are also consistent with recent findings showing an association between verbal working memory capacity and musicianship (29)

### Anticipation

Previous research showed no practice effect on EHS (see Eye-hand span section); this is confirmed by the present study if we consider a conservative criterion for statistical adjustment. Nevertheless, we observed a practice effect on the awareness span, which in turn was significantly correlated with the EHSQ in the PCA. Furthermore, the EHS learning curve was gradual (non-monotonic) throughout the trials, reaching its maximum increase in the third trial and decreasing in the fourth.

In terms of the musical structure, the increase in the EHS was particularly decisive in those sections presenting thematic development (which may sound counterintuitive). This indeed suggests that pianists' anticipation strategies were critically oriented, stressing the relationship between music structure and anticipation. These results confirm our hypotheses concerning the effect of music structure on EHS, and partially confirm the effect of practice on anticipation.

The awareness span can be considered a measure of expertise which behaves in general like the EHS. In this context, and taking into account our results, the planning of motor movements can be improved during a short session. Moreover, this improvement could be related to the ability to plan ahead segment-by-segment, which depends on working memory capacity to keep up with real-time music performance speed (11). Our results suggest that improvement in the awareness span in a short session is linked with more effective control of planning strategies and knowledge-based expectations, allowing pianists to maximize anticipation in particular sections of the score selectively and progressively until they reach the limit of their capabilities. This appears to be consistent with the results of Lim et al. (55) in sight-reading performances.

In fact, it is interesting to note that the EHSQ was one of the determining variables in the PCA, probably because it is the most regular rhythmic figure throughout the piece (in comparison with the other measurements of the EHS). These results confirm our first hypothesis and highlight the importance of establishing different expertise approaches, accounting for individual differences in learning a new piece of music. In fact, the systematic review of Perra et al. (67) points out that the criteria used to assign musicians to an expertise group are not consistent in the literature, which makes it difficult to compare the different studies.

Future research should be conducted isolating specific issues related to the visual monitoring of motor movements and combining the EHS and the awareness span measures.

In short, anticipation by the musician is a vital part of music reading. Furthermore, prediction mechanisms occur at different structural levels (65), determining the involvement of different cognitive substrates to ensure anticipation, which, according to our results, would be mediated by expertise.

### Learning and Skill

We believe that our decision not to impose a specific tempo was appropriate as it allowed the pianists to follow their own pace of learning throughout the trials. Indeed, imposing a tempo after a first or second reading on semi-professional or professional musicians in an individual reading situation does not make much sense in our opinion. On the other hand, our results are consistent with previous research on music reading using eye-tracking techniques. However, we understand that there are different positions on the matter (43; 55; 66). We believe that one relevant finding of this research is to show that both approaches are complementary and not exclusive, given the complexity of the learning process and the multiplicity of musical styles and factors that can influence the process of learning a new piece of music.

Expertise is associated with fewer GAK. In parallel we observed an increase of GAK in those sections that present thematic development. These results seem to fit with early intuitions about GAK, suggesting that “the quality of the movements is more important than the quantity” (49, p. 97). In turn, the reduction in information acquisition associated with expertise, and explained by the parallel decrease in the number of fixations, could result from the operationalization of representations, with a consequent reduction in the number and duration of GAK. Moreover, the duration of GAK decreased in the final section, which presents an ostinato. The foregoing suggests that thematic development would be associated with a decrease in the duration of the GAK, which is reinforced by the idea of the ostinato since both elements point towards independence from the keyboard as part of expert behaviour.

The limitations of the present research relate to working with naturalistic musical material and the use of free tempo. However, these limitations could also be a strength as we could observe different approaches to music learning and more cross-cutting strategies that could be assessed with different expertise approaches.

### Conclusions

In our view, musicians use different strategies that vary according to their degree of expertise, reflected in oculomotor and anticipation behaviour. These observations are consistent with the findings of Lim et al. (55), who studied the link between music complexity and anticipation (see also 71). Everything seems to indicate that a dynamic relationship is established between the different variables throughout the learning process, where periods of instability alternate with periods of stability (see 3); according to Timmers et al. (80), this is an indicator of skill acquisition. Indeed, learning is achieved through music structure as an important cue for improving performance and refining learning strategies in the initial phase of learning, consistent with previous findings (14; 15; 86).

To summarize, using eye-tracking techniques to study the early learning process in professional and advanced undergraduate pianists brings us new perspectives about how musicians approach an unfamiliar piece. The effect of the structure of the music is conclusive, resulting in the development of different learning and planning strategies. These learning mechanisms could lead, for example, to more effective anticipation strategies and a selective mobilization of working memory resources.

### Ethics and Conflict of Interest

The author declares that the contents of the article are in agreement with the ethics described in http://biblio.unibe.ch/portale/elibrary/BOP/jemr/ethics.html and that there is no conflict of interest regarding the publication of this paper.

### Acknowledgements

This research was supported in part by grants from the National Commission for Scientific and Technological Research (CONICYT) N° 72100347.

I would like to express my gratitude to Dr. Paul Molin for his feedback and support.
